# Transcriptomic Analysis of Gonadal Adipose Tissue in Male Mice Exposed Perinatally to 2,2′,4,4′-Tetrabromodiphenyl Ether (BDE-47)

**DOI:** 10.3390/toxics6020021

**Published:** 2018-03-29

**Authors:** Aser Abrha, Alexander Suvorov

**Affiliations:** Department of Environmental Health Sciences, University of Massachusetts Amherst, 686 N. Pleasant St., 149A Goessmann, Amherst, MA 01003, USA; aser.abrha@gmail.com

**Keywords:** polybrominated diphenyl ether, PBDE, BDE-47, adipose, transcriptomic, genomic, obesogen, complement and coagulation cascade, de novo lipogenesis, metabolism

## Abstract

For the majority of lipophilic compounds, adipose tissue is traditionally considered as a storage depot and only rarely as a target organ. Meanwhile, abnormalities in adipose tissue physiology induced by chemical exposure may contribute to the current epidemic of obesity and metabolic diseases. Polybrominated diphenyl ethers (PBDEs) are a group of lipophilic flame retardants found in the majority of human samples in North America. Their ability to alter the physiology of adipose tissue is unknown. We exposed pregnant mice to 0.2 mg/kg body weight/day of BDE-47 perinatally. Transcriptomic changes in gonadal adipose tissue were analyzed in male offspring using the RNA-seq approach with subsequent bioinformatic analysis. The expression of genes of coagulation and complement cascade, de novo lipogenesis, and xenobiotic metabolism was altered in response to BDE-47 exposure. The affected molecular network included the following hubs: PPARα, HNF1A, and HNF4. These findings suggest that adipose tissue should be considered a target tissue for BDE-47, in addition to its role as a storage depot. This study also builds a background for a targeted search of sensitive phenotypic endpoints of BDE-47 exposure, including lipid profile parameters and coagulation factors in circulation. Additional studies are needed to investigate the role of PBDEs as an obesogen.

## 1. Introduction

Metabolic disease represents one of the biggest public health challenges today. Approximately two out of every five Americans will develop type 2 diabetes at some point during their adult lives [1]. More than 60% of children above the age of 10 either are obese or will become obese later in life. One in four overweight children suffers from impaired glucose tolerance [2]. A growing body of evidence links the current worldwide epidemics of obesity, diabetes, and related metabolic diseases with environmental exposure [3]. Many environmental xenobiotics, such as dioxins, PCBs, PBDEs, and others, are highly lipophilic and therefore accumulate in adipose tissue in humans, livestock, and wildlife. Adipose tissue is usually considered a storage depot of lipophilic compounds and is only rarely considered a target of toxicity in toxicological research [4], although it is known to be involved in several physiological functions, including metabolic regulation, energy storage, and endocrine functions [4]. For example, white adipose tissue is a major secretory cell of a diverse list of proteins collectively known as adipokines involved in the regulation of metabolic functions of the organism [5]. Changes in adipose physiology induced by environmental xenobiotics may be associated with obesity, diabetes, and other metabolic diseases [6,7].

Polybrominated diphenyl ethers (PBDEs) are a type of brominated flame retardants that were used in many household products such as paints, plastics, textiles, furniture padding, mattresses, and various electrical appliances until recently [8]. Although the commercial use of PBDEs has ceased in many industrial countries, PBDEs are ubiquitously found in the environment due to their resistance to degradation. The level of PBDEs in human blood, breast milk, and adipose tissue has increased exponentially over the last three decades and continues to rise in North America [9]. Among the various PBDE congers, 2,2′,4,4′-tetrabromodiphenyl ether (BDE-47) is the most prevalent in human samples, including maternal milk [10,11,12,13,14,15]. PBDEs are lipophilic and bioaccumulate in fatty tissues with a half-life in human tissues estimated at 1.8–6.5 years [16]. Although PBDEs accumulate predominantly in adipose tissue, the majority of studies of PBDE toxicity were focusing on neurodevelopment, endocrine, and reproductive endpoints [17,18,19] and target organs such as the liver, thyroid gland, kidney, and reproductive organs. The ability of PBDEs to affect the physiology of adipose tissue was not thoroughly addressed in previous studies.

In this study, we analyze global gene expression in gonadal adipose tissue of mice exposed perinatally to a low dose (0.2 mg/kg body weight) of BDE-47. Our data demonstrate significant changes in several functional networks including coagulation and complement cascade, lipid and lipoprotein synthesis, and xenobiotic metabolism pathways.

## 2. Materials and Methods

### 2.1. Animals and Treatment

Eight-week-old male (30–35 g) and female (27–30 g) CD-1 mice were obtained from Charles River Laboratories (Kingston, NY, USA) and housed in a temperature (23 ± 2 °C)- and humidity (40 ± 10%)-controlled environment, with a 12-h light/dark cycle, and food and water available ad libitum. After three days of acclimation, the animals were bred and the day of vaginal plug detection was considered pregnancy day 1. The dams were assigned to one of two treatment groups (*n* = 5 per group) based on weight match, and were exposed to tocopherol-stripped corn oil (MP Biomedicals, Solon, OH, USA) or a 0.2 mg/mL solution of BDE-47 (AccuStandard, Inc., New Haven, CT, USA; 100% purity) in tocopherol-stripped corn oil daily from pregnancy day 8 through postpartum day 21; the females were fed 1 µL/g BW from the tip of a pipette, resulting in exposure of 0.2 mg/kg BW/day. This method of exposure is routinely used in our laboratory as a substitution of oral gavage as the latter method induces significant stress response by the endocrine system, which may interfere with the analyzed health outcomes [20]. The dams were allowed to deliver naturally, and the litters were not culled to maintain consistency of nutrient distribution among the same number of fetuses/pups at pre- and postnatal periods, and to avoid catch-up growth [21]. The dams and pups were kept together until weaning on PND 21, when the male and female pups were separated. Using cervical dislocation, one randomly selected male pup per litter was euthanized on PND21. The other pups were used in a different study. All animals were euthanized between 9 a.m. and 11 a.m., following 2 h of fasting. Gonadal fat from the right side of each animal was collected, weighed, and snap-frozen in liquid nitrogen and stored at −80 °C. All procedures met the guidelines of the National Institutes of Health Guide for the Care and Use of Laboratory Animals, and this study was approved by the Institutional Animal Care and Use Committee at University of Massachusetts, Amherst, MA, USA.

### 2.2. RNA Extraction and Sequencing

Four samples of gonadal fat per exposure group were used for RNA extraction, collected from four individual pups from four different litters per exposure group. Total RNA was isolated using TRIzol reagent (Invitrogen, Carlsbad, CA, USA) and quantified using a NanoDrop 1000 instrument (Thermo Fisher Scientific, Wilmington, DE, USA). RNA quality was assessed using Agilent 2100 Bioanalyzer (Agilent Technologies, Santa Clara, CA, USA). Samples of RNA with integrity values > 9 were used for library preparation in accordance with the recommendations of the library preparation and sequencing kit manuals. The NEBNext Poly(A) mRNA Magnetic Isolation Module (E7490, New England BioLabs Inc., Ipswich, MA, USA) was then used to isolate intact poly(A) + RNA from 3 µg of total RNA. Libraries were constructed using NEBNext mRNA Library Prep Reagent Set for Illumina with multiplexing indexes from NEBNext Multiplex Oligos for Illumina (E6100 and E7335 respectively, New England BioLabs Inc., Ipswich, MA, USA). The quality and purity of the libraries were assessed by Agilent 2100 Bioanalyzer, and the concentration of the libraries was measured by real-time PCR with primers for P5 and P7 flow cell oligo sequences using a KAPA Library Quantification Kit (KR0405, Kapa Biosystems, Boston, MA, USA) in a 384-well plate on a CFX384 Touch Real-Time PCR Detection System (Bio-Rad, Hercules, CA, USA). High-throughput sequencing was performed on a NextSeq500 sequencing system (Illumina, San-Diego, CA, USA) in the Genomic Resource Laboratory of the University of Massachusetts, Amherst. cDNA libraries were single-end sequenced in 75 cycles using NextSeq 500 High Output Kit (FC-404-1005, Illumina, San-Diego, CA, USA) in a multiplex run. Four samples were sequenced per exposure group, representing eight different litters. Sequencing was completed with minimum 20 million reads per sample. One control sample was sequenced with smaller number of reads and was excluded from further analysis. All sequencing data were uploaded to the GEO public repository and GEO were assigned series accession number GSE112207.

### 2.3. RT-qPCR

The RNA-seq results were validated using RT-qPCR for genes selected randomly from the list of the most significantly differentially regulated genes in RNA-seq experiment (Table 1). Total RNA was purified of genomic DNA contamination using DNase (RQ1 RNAse-free DNAse, Cat. # M610A, Promega, Madison, WI, USA) and reverse-transcribed using the High Capacity cDNA Reverse Transcription Kit (Cat. # 4368814, Applied Biosystems, Vilnus, Lithuania). Using free online software Primer3Plus (primer3plus.com), forward and reverse primers were designed to anneal different exons spanning long intron (Table 1). The housekeeping gene (Polr3c) was selected from genes that were not regulated in our RNA-seq dataset, with the consideration of its ubiquitous presence in different cell types. Triplicate 5-μL real-time PCR reactions, each containing iTaq Universal SYBR Green Supermix (Cat. 172-5124, BioRad), primers, and cDNA template were loaded onto a 384-well plate and run through 40 cycles on a CFX384 real time cycler (Bio-Rad Laboratories, Inc.). The data were analyzed using the manufacturer’s CFX manager software (version 3.1, BioRad, Hercules, CA, USA, 2013). Relative quantification was determined using the ΔΔCq method [22].

### 2.4. Bioinformatic Analysis of RNA-seq Data

To analyze the data from our mRNA sequencing experiment, read filtering, trimming and de-multiplexing were performed via the BaseSpace cloud computing service supported by Illumina (https://basespace.illumina.com/home/index). The preprocessed reads were mapped to the reference mouse genome (MM10) using TopHat 2 aligner [23,24]. Aligned reads were then used for assembly of novel transcripts with Cufflinks 2.1.1 and differential expression of novel and reference transcripts with Cuffdiff 2.1.1 [25]. The list of differentially expressed transcripts was filtered to select only transcripts associated with gene names and transcripts with FPKM values > 1 in at least one of conditions (control or exposed). The resulting gene list included 14,289 genes.

DAVID bioinformatics resources version 6.7, Gene Set Enrichment Analysis (GSEA), and Ingenuity Pathway Analysis (IPA) were used for bioinformatics analysis of RNA-seq data. A short list of 2-fold significantly differentially expressed genes was imported to DAVID or IPA for functional analysis with default settings. GSEA analysis was conducted using a ranked list of 14,289 genes against the following collections of gene sets: hallmark genes sets (H), curated gene sets from online pathway databases, publications in PubMed, and knowledge of domain experts (C2) and gene ontology gene sets (C5) obtained from the molecular signatures database v5.2 [26] and PPARα target genes obtained from [27]. Gene sets enriched with FDR *q*-value < 0.02 were considered significant.

## 3. Results

We found no significant relationship between litter size and exposure to BDE-47, with the number of pups varying from 11 to 15 per litter. No weight differences were observed between the control and exposed dams and pups throughout the experiment. No changes in adipose tissue weight were found in male mice on PND21.

**Figure 1:**
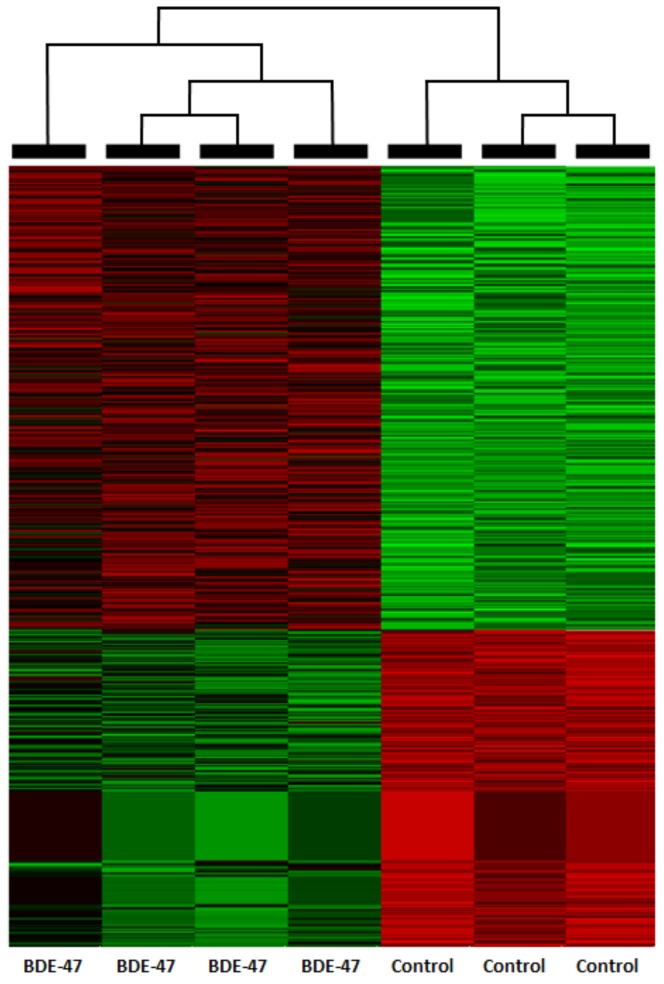
Heat-map for 758 2-fold differentially regulated genes in mouse gonadal adipose tissue on PND21.

The resulting filtered list of differentially expressed genes included 14,289 genes. 758 genes were deferentially expressed 2-fold or higher (Figure 1) and 94 genes also had FDR *q*-value of ≤ 0.05 (Table S1). Expression of select genes measured by RT-PCR showed good concordance with RNA-seq data (Table 1).

### 3.1. DAVID Analysis

**Figure 2:**
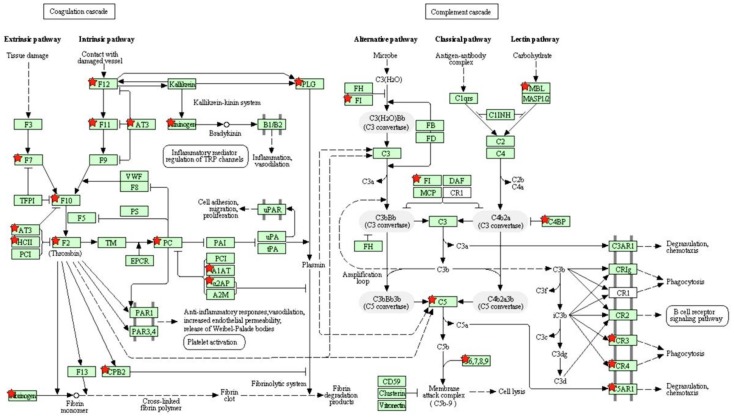
KEGG coagulation and complement cascades pathway. Differentially expressed genes are marked with red stars.

DAVID analysis revealed four enriched annotation clusters with an enrichment score >2.0: ‘secreted proteins’, ‘peptidase inhibitor activity’, ‘complement and coagulation cascades’ (Figure 2), ‘biosynthesis of amino acid’, ‘microsomal metabolism’, and ‘lipid metabolism’ (Table 2).

### 3.2. GSEA Analysis

**Figure 3:**
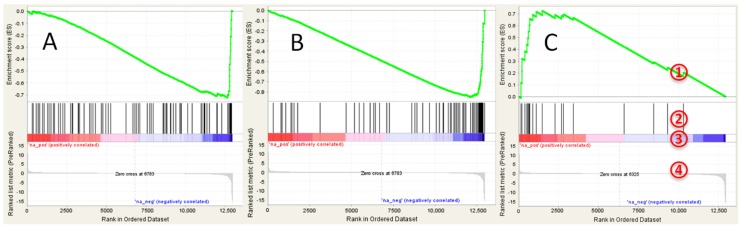
GSEA enrichment plots for transcriptional changes in gonadal adipose tissue of mice exposed perinatally to BDE-47: (**A**) bile acid metabolism gene set; (**B**) genes targets of HNF4A; and (**C**) genes targets of SREBF. GSEA plot legend: 1—running enrichment score for the gene set; 2—vertical lines show where the members of the gene set appear in the ranked list of genes; 3 and 4—ranked list of differentially expressed genes from the most up-regulated (left of each plot) to the most downregulated (right of each plot). Regulation is shown in the heat-bar (3) and in the bar plot (4).

No significantly positively enriched gene sets were found in GSEA analysis (see Table S2 for details). The following hallmark gene sets were negatively enriched with FDR *q* < 0.2: ‘coagulation’, ‘xenobiotic metabolism’, and ‘bile acid metabolism’ (Figure 3A). Analysis of C2 collection of gene sets revealed several negatively regulated liver specific gene sets, including genes targets of HNF1A and HNF4A (Figure 3B). Several positively enriched (although not significant) gene sets indicated upregulation of lipid biosynthesis, including ‘SREBF targets’ (Figure 3C), ‘obesity’ and ‘cholesterol biosynthesis’. Significantly negatively enriched gene ontology (GO) gene sets included ‘coagulation’, ‘serine type endopeptidase activity’, and ‘lipid catabolism’. Genes—targets of PPARα alpha were also significantly downregulated (normalized enrichment score = −1.72, nominal *p*-value < 0.0001, FDR *q* < 0.001).

### 3.3. IPA Analysis

**Figure 4:**
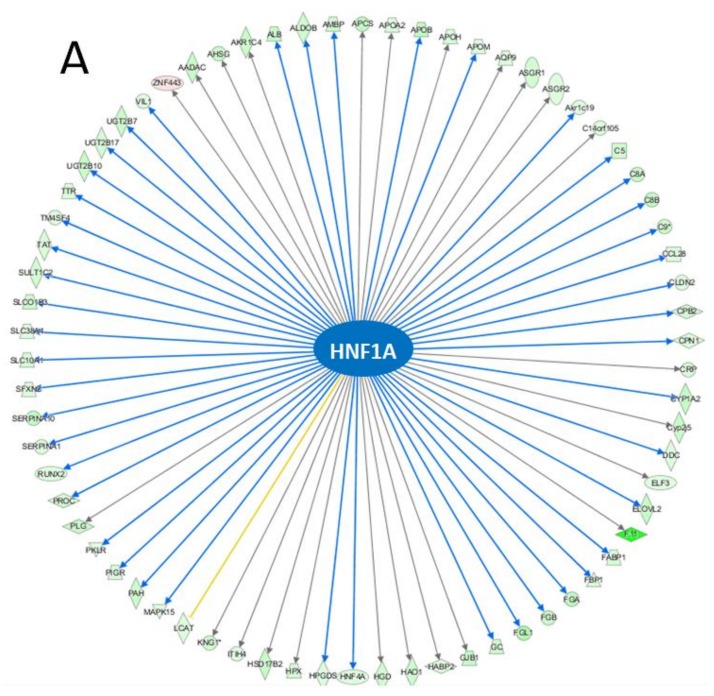

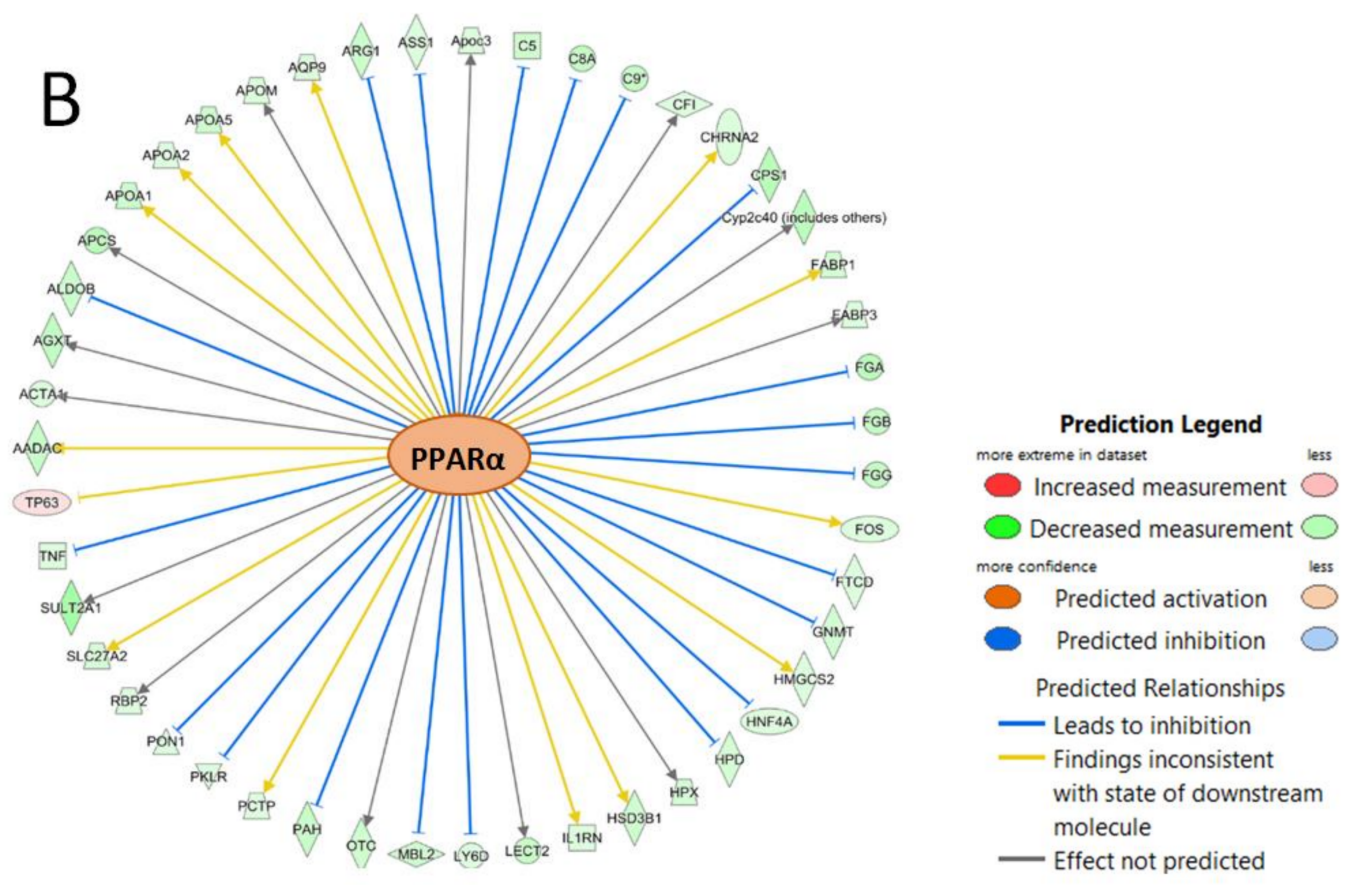
Molecular networks of differentially expressed genes regulated by (**A**) HNF1A and (**B**) PPARα.

The top IPA enriched canonical pathways were ‘FXR/RXR activation’, ‘LXR/RXR activation’, ‘acute phase response signaling’, ‘coagulation system’, and ‘extrinsic prothrombin activation pathway’ (Table 3). HNF1A (*p*-value of overlap = 2.98 × 10^−37^, predicted activation—inhibited), and PPARα (*p*-value of overlap = 1.41 × 10^−18^, predicted activation—n/a) were identified as upstream regulators with significantly altered activity (Figure 4).

## 4. Discussion

We found that perinatal exposure to 0.2 mg/kg body weight of BDE-47 results in changes in the expression of genes involved in the coagulation and complement cascades, xenobiotic metabolism and lipid metabolism in gonadal adipose tissue of mice, suggesting that adipose tissue can also be a target tissue for BDE-47, in addition to its role as a storage depot.

### 4.1. Relevance of the Dosing Paradigm

Results reported in this study were produced using environmentally-relevant exposure paradigm. In our previous study [28], exposure of pregnant rats to 0.2 mg/kg body weight of BDE-47 resulted in 234.3 ng BDE-47/g lipid in adipose tissue of dams and 1054.7 ng BDE-47/g lipid in adipose tissue of pups. These concentrations are comparable with those of the North American human population (mean concentration in adipose tissue of adult subjects = 399 ng/g lipids) [29]. Given that the rate of BDE-47 elimination is around 10 times higher in mice than in rats [30,31], the exposure used in this study is also relevant to that of the North American human population. In this study we dosed animals during the pre- or neonatal period since human PBDE exposure peaks during the perinatal period of life due to the active transport of PBDE via cord blood and breast milk [32,33,34], higher rates of dust ingestion [35], and higher food intake per kilogram of body weight in toddlers [33].

### 4.2. Downregulation of the Complement and Coagulation Cascades Genes

Coagulation is the primary defense against blood loss at a site of injury. It is characterized by a serine protease mediated conversion of zymogens to their active form to induce blood clotting [36]. The major players in the coagulation and fibrinolytic system are circulating zymogens (fibrinogen, clotting factors, protein C and its cofactor protein S, plasminogen, kininogen), activating and converting enzymes (plasminogen and thrombin), a group of serine protease inhibitors (SERPINs), and high density lipoproteins (HDL). Our results indicate that the expression of the major players in the coagulation pathway and fibrinolytic pathway, namely fibrinogen protein chains (*FGG*, *FGA*, *FGB*), clotting factors (*F2*, *F7*, *F10*, *F11*, and *F12*), serine protease inhibitors (*SERPINC1*, *SERPIND1*, *SERPINF2*), plasminogen (*PLG*), and protein C (*PROC*) were downregulated by more than 2-fold. We also observed more than 2-fold downregulation of the complement cascade genes such as *C9*, *C8A*, *C8B*, *C4bp Cfi*, *HC*, *Mbl1*, and *MBl2*. Adipose tissue has an important role in direct regulation of the coagulation and complement system by producing coagulation tissue factors and complement proteins [37,38]. Therefore, the observed changes in gene expression may have significant consequences for the whole complement and coagulation system in exposed organisms.

### 4.3. Downregulation of Xenobiotic Metabolism Genes

A big group of heterogeneous xenobiotic metabolism genes, including but not limited to cytochromes P450 (Cyp1a, Cyp2c, and Cyp3a subfamilies), was downregulated in exposed mice. This group of genes is usually induced in toxicological experiments, including experiments with BDE-47 [39,40,41,42,43], while in our experiment enrichment was negative, corresponding to a significant decrease in the expression of many genes. We speculate that this may be a result of overcompensation for sub-chronic developmental exposure to a low dose of BDE-47. Similarly, changes in gene expression, opposite in direction to predicted, were reported recently for liver tissue in BDE-47-exposed mice [44]. Altered expression of P450 cytochrome genes may affect the availability of active forms of vitamin D [45], which was recently shown to regulate the adipogenic fate of adipose-derived stem cells [46].

### 4.4. Upregulation of De Novo Lipogenesis Genes

Our bioinformatic analysis suggests that the metabolic profile of adipose tissue in exposed animals is slightly shifted in the direction of activation of de novo lipogenesis (DNL). For example positive enrichment of GSEA ‘*SREBF targets’* dataset was due to upregulation of several essential DNL genes, including 1.6-fold induction of Acaca, encoding DNL rate-limiting enzyme acetyl-CoA carboxylase [47]. Genes of cholesterol biosynthesis were also upregulated, including 1.5-fold induction of Hmgcs1, encoding cytosolic 3-hydroxy-3-methylglutaryl-CoA (HMG-CoA) synthase, a rate-limiting enzyme of cholesterol biosynthesis [48]. At the same time, bile acid metabolism genes were downregulated, suggesting decreased cleavage of cholesterol, concordant with the previously reported increase in circulating cholesterol in rats perinatally exposed to BDE-47 [39]. However, it is difficult to interpret the observed changes in molecular pathways responsible for cholesterol turnover in the current study, as adipose tissue is not a major player in cholesterol biosynthesis and elimination. Importantly, changes in gene expression discussed in this paragraph may affect the major function of the adipose tissue—to store energy in the form of lipids. This concern is supported by human population data reporting a positive association between PBDE concentrations in blood, milk, and placenta and BMI in nursing women [34,49,50,51]. In several animal studies, developmental BDE-47 exposures also resulted in increased body weight [52,53,54].

### 4.5. Network Affected by BDE-47

The coagulation and complement system is known to be regulated among other transcription factors by HNF1, HNF4α and PPARα—mechanisms enriched in our bioinformatic analysis. For example the α and β promoters of fibrinogen gene contain tissue specific motif for HNF1 [55]. SERPINC1 (antithrombin) is transcriptionally activated by the binding of HNF1α and HNF4α to SERPINC1 gene (*α1AT*) promoter/enhancer [56]. Additionally, synthetic PPARα agonists, fibrates, have been shown to suppress fibrinogen gene expression and impair the induction of tissue factors in endothelial cells in rodents [57,58] and downregulate the complement and coagulation pathways in cynomolgus monkey liver [59]. Both transcription factor HNF4A and PPARα regulate expression of major protein component of the high density lipoproteins (HDL) [60,61]. In mice (contrary to humans), PPARα upregulates expression of *APO5*, *APOA1*, and *APOA2* [62,63,64]. In our study, the expression of several HDL genes *APOA1*, *APOA2*, *APOA5*, *APOB*, *APOC2*, *APOC3*, *APOC4*, *APOH*, *APOM* was more than 2-fold downregulated. HDL modulates protease activity by directly binding to the majority of SERPINs [65]. Finally the expression of PPARα is regulated by HNF4 in mice, and PPARα is capable of binding to HNF4αRE [66].

### 4.6. Role of PPARα in the Modulation of BDE-47 Induced Response

Our analysis identifies PPARα as a hub of a molecular network affected in adipose tissue by BDE-47. No studies reported previously BDE-47 interaction with PPARα. In fact, a theoretical structure–activity assessment predicted that PBDEs would not have an interaction with PPAR receptors [67]. However, several studies have indicated the ability of BDE-47 and/or its metabolites to activate another PPAR isoform—PPARγ [68,69,70]. This evidence, taken together with the fact that the PPAR nuclear family is considered to have the largest and the most promiscuous binding pocket [71], suggests the possibility that most of the observed changes in gene expression in the current study are a result of direct interaction of BDE-47 and/or its metabolites with PPARα. An alternative hypothesis may explain the changes in PPARα activity by the ability of BDE-47 to interact with regulatory mechanisms upstream of the receptor. In our recent study we have shown that the same dosing paradigm used in this study results in activation of mTOR signaling in mouse livers on PND21 [44]. PPARα is a well-known target of mTOR complex 1 (mTORC1) [72,73,74]. Additional studies are needed to determine the interaction between BDE-47 and the PPAR-regulated molecular network.

## 5. Conclusions

We have demonstrated that low-dose perinatal exposure to BDE-47 produces significant changes in gene expression in the gonadal adipose tissue in mice, suggesting that adipose tissue should be considered a target tissue for BDE-47, in addition to its role as a storage depot. Our data imply that functional networks, including coagulation and complement cascades, de novo lipogenesis, and xenobiotic metabolism, are affected by BDE-47. The affected molecular network is connected via the following hubs: PPARα, HNF1A, and HNF4. Altered metabolism of vitamin D due to the changes in P450 gene expression may interfere with adipose tissue function. This study builds a background for a targeted search of sensitive phenotypic endpoints of BDE-47 exposure, including lipid profile parameters and coagulation factors in circulation. Additional research is needed to dissect molecular cascades affected in the adipose tissue by PBDEs, and the role of PBDEs as obesogens.

## Figures and Tables

**Table 1:** RT-qPCR validation of RNA-seq data for select differentially expressed genes.

Gene	qPCR Primers	Fold Change	
		RNA-seq	qPCR
Grem2	GAGGAGAGGGACAGGGAGAC	2.8	2.6
	AGCGAGAGCTTCCAGAACAT		
Cish	CGTTGTCTCTGGGACATGGT	4.4	3.9
	ACAGCCAGCAAAGGACAAGA		
Sfrp2	AGGACAACGACCTCTGCATC	4.3	4.2
	TTCTTGGTTTTGCAGGCTTC		
Fgg	AACCCACGAGACAAGCATTC	−15.7	−16.3
	CAAGCTGGGCTACCTTCTGT		
F2	CCCTGGTTAGCCTGGTACAC	−16.2	−13.9
	CAGCGATAGTGCTTGCTGAG		
Apob	AAAACTTAAGCTTCAGCTGTCCA	−20.3	−17.5
	CTTTCGGAGGTGCTTGAATC		
B2m	CCGGCCTGTATGCTATCCAG	--	housekeeping
	TGTTCGGCTTCCCATTCTCC		

**Table 2:** DAVID functional annotation clustering for 94 2-fold differentially expressed genes (FDR *q* ≤ 0.05).

Functional Annotation Cluster	Enrichment Score	Genes	*p*-Value	Benjamini
*Secreted proteins*	11.17	38	6.7 × 10^−6^–3.0 × 10^−18^	7.0 × 10^−4^–5.9 × 10^−16^
*Peptidase inhibitor activity*	5.15	10	2.7 × 10^−2^–1.9 × 10^−9^	6.2 × 10^−1^–9.2 × 10^−8^
*Blood coagulation*	3.14	22	2.7 × 10^−2^–1.9 × 10^−9^	6.2 × 10^−1^–9.2 × 10^−8^
*Biosynthesis of amino acids*	2.81	6	4.6 × 10^−2^–1.2 × 10^−4^	3.0 × 10^−1^–5.7 × 10^−3^
*Microsomal metabolism*	2.40	7	1.1 × 10^−1^–1.9 × 10^−5^	5.3 × 10^−1^–4.5 × 10^−4^
*Lipid metabolism*	2.27	5	3.8 × 10^−2^–7.8 × 10^−4^	3.9 × 10^−1^–3.0 × 10^−2^

**Table 3:** Ingenuity Pathway Analysis: top enriched canonical pathways.

Canonical Pathway	*p*-Value	Overlap	Count
*FXR/RXR Activation*	2.4 × 10^−^^23^	25.4%	32/126
*LXR/RXR Activation*	2.99 × 10^−^^17^	21.5%	26/121
*Acute Phase Response Signaling*	3.02 × 10^−^^17^	17.8%	30/169
*Coagulation System*	1.71 × 10^−^^15^	42.9%	15/35
*Extrinsic Prothrombin Activation Pathway*	3.68 × 10^−^^11^	56.2%	9/16

## Supplementary Materials

The following are available online at http://www.mdpi.com/2305-6304/6/2/21/s1, Table S1: Significant 2-fold differentially expressed genes in gonadal adipose tissue of mice exposed perinatally to 0.2 mg/kg body weight BDE-47; Table S2: GSEA analysis: summary of top enriched gene sets in the gonadal adipose tissue of mice exposed perinatally to 0.2 mg/kg body weight BDE-47.
