# Supplementary material for: Transcriptomic Analysis of Gonadal Adipose Tissue in Male Mice Exposed Perinatally to 2,2′,4,4′-Tetrabromodiphenyl Ether (BDE-47)
Source: Toxics. 2018 Mar 29;6(2):21. doi: 10.3390/toxics6020021 (PMC6027300; doi:10.3390/toxics6020021)
Supplement: Supplementary file 1 [file toxics-06-00021-s001.pdf]

# Supplementary Materials: Transcriptomic Analysis of Gonadal Adipose Tissue in Male Mice Exposed Perinatally to 2,2′,4,4′-Tetrabromodiphenyl Ether (BDE-47)

**Aser Abrha and Alexander Suvorov**

**Table S1.** Significant 2-fold differentially expressed genes in gonadal adipose tissue of mice exposed perinatally to 0.2 mg/kg body weight BDE-47.

| Gene | Locus | log2 (Control FPKM) | log2 (BDE-47 FPKM) | log2 (Ratio) | *q* Value |
|---|---|---|---|---|---|
| Kif27, Mir6369 | chr13:58287515-58354862 | −0.91 | 3.35 | 4.26 | $2.21 \times 10^{-2}$ |
| Mir3061 | chr11:52123633-52127767 | −0.08 | 3.66 | 3.73 | $1.30 \times 10^{-2}$ |
| A330023F24Rik, Mir29b-2, Mir29c | chr1:195017157-195039001 | 1.51 | 4.66 | 3.15 | $1.30 \times 10^{-2}$ |
| Actg2 | chr6:83512908-83536251 | 0.63 | 3.76 | 3.12 | $1.30 \times 10^{-2}$ |
| Mir17hg, Mir19b-1 | chr14:115044304-115046851 | 0.54 | 2.85 | 2.3 | $4.75 \times 10^{-2}$ |
| Cish | chr9:107296688-107301961 | 1.12 | 3.25 | 2.13 | $1.30 \times 10^{-2}$ |
| Sfrp2 | chr3:83766320-83774314 | 2.38 | 4.48 | 2.1 | $1.30 \times 10^{-2}$ |
| Epc1, Mir1893 | chr18:6435950-6516108 | 3.12 | 4.64 | 1.52 | $1.30 \times 10^{-2}$ |
| Grem2 | chr1:174833784-174921819 | 1.78 | 3.29 | 1.51 | $1.30 \times 10^{-2}$ |
| Mir5122, Slc9a1 | chr4:133369771-133423698 | 3.48 | 4.84 | 1.36 | $1.30 \times 10^{-2}$ |
| Axin2 | chr11:108920348-108950785 | 2.8 | 4.09 | 1.29 | $1.30 \times 10^{-2}$ |
| Myh11 | chr16:14194364-14291408 | 3.9 | 5.18 | 1.28 | $1.30 \times 10^{-2}$ |
| Tpm2 | chr4:43513725-43523554 | 4.27 | 5.31 | 1.04 | $1.30 \times 10^{-2}$ |
| Mpeg1, Pfpl | chr19:12427904-12465285 | 5.76 | 4.7 | −1.06 | $1.30 \times 10^{-2}$ |
| Cadm1 | chr9:47530351-47853385 | 3.02 | 1.91 | −1.11 | $3.19 \times 10^{-2}$ |
| Ctsk | chr3:95499209-95509387 | 4.65 | 3.52 | −1.13 | $1.30 \times 10^{-2}$ |
| Nceh1 | chr3:27183003-27244911 | 3.33 | 2.2 | −1.14 | $1.30 \times 10^{-2}$ |
| Apba3, Mir3057 | chr10:81267855-81291581 | 4.83 | 3.67 | −1.16 | $1.30 \times 10^{-2}$ |
| Mmp12 | chr9:7347373-7360461 | 6.58 | 5.42 | −1.16 | $1.30 \times 10^{-2}$ |
| Ccl9 | chr11:83572916-83578636 | 5.58 | 4.4 | −1.18 | $1.30 \times 10^{-2}$ |
| Trpv2 | chr11:62574485-62600467 | 5.07 | 3.85 | −1.23 | $1.30 \times 10^{-2}$ |
| Ccl6 | chr11:83582060-83623693 | 7.45 | 6.21 | −1.24 | $1.30 \times 10^{-2}$ |

| | | | | | |
|---|---|---|---|---|---|
| Cd300lb | chr11:114922778-114934386 | 4.04 | 2.8 | −1.24 | $4.75 \times 10^{-2}$ |
| Atf3 | chr1:191170296-191183333 | 4.5 | 3.23 | −1.28 | $1.30 \times 10^{-2}$ |
| Arhgap25 | chr6:87458544-87533259 | 4.09 | 2.81 | −1.28 | $2.21 \times 10^{-2}$ |
| Ubd | chr17:37193891-37196101 | 6.15 | 4.87 | −1.28 | $4.75 \times 10^{-2}$ |
| Blnk | chr19:40928926-40994535 | 3.78 | 2.48 | −1.3 | $4.07 \times 10^{-2}$ |
| Bok | chr1:93685693-93695762 | 4.78 | 3.44 | −1.34 | $2.21 \times 10^{-2}$ |
| Cd300a | chr11:114890040-114906474 | 3.2 | 1.84 | −1.36 | $1.30 \times 10^{-2}$ |
| Cd52 | chr4:134093537-134095073 | 6.59 | 5.23 | −1.36 | $1.30 \times 10^{-2}$ |
| Lgals3 | chr14:47373859-47386167 | 8.2 | 6.83 | −1.37 | $1.30 \times 10^{-2}$ |
| Thbs1 | chr2:118111838-118127139 | 7.4 | 5.98 | −1.41 | $1.30 \times 10^{-2}$ |
| Specc1 | chr11:61956762-62223013 | 2.8 | 1.35 | −1.44 | $1.30 \times 10^{-2}$ |
| Atp6v0d2 | chr4:19876837-19922566 | 4.84 | 3.36 | −1.48 | $1.30 \times 10^{-2}$ |
| C5ar1 | chr7:16246742-16259540 | 4.54 | 3.05 | −1.49 | $1.30 \times 10^{-2}$ |
| Lcn2 | chr2:32384636-32387739 | 5.85 | 4.31 | −1.54 | $1.30 \times 10^{-2}$ |
| Hmgcs2 | chr3:98280430-98310738 | 5.12 | 3.58 | −1.54 | $1.30 \times 10^{-2}$ |
| Rnf128 | chrX:139563339-139673145 | 3.29 | 1.63 | −1.67 | $2.21 \times 10^{-2}$ |
| Mt2 | chr8:94172617-94173567 | 9.84 | 8.12 | −1.71 | $1.30 \times 10^{-2}$ |
| Slc38a3 | chr9:107651154-107668968 | 3.49 | 1.78 | −1.71 | $1.30 \times 10^{-2}$ |
| Ass1 | chr2:31470269-31520670 | 4.33 | 2.5 | −1.83 | $1.30 \times 10^{-2}$ |
| Mir6537, Pou2f2 | chr7:25090634-25132462 | 2.71 | 0.65 | −2.06 | $1.30 \times 10^{-2}$ |
| Acta1 | chr8:123891757-123894775 | 6.91 | 4.84 | −2.07 | $1.30 \times 10^{-2}$ |
| Itih4 | chr14:30886475-30901986 | 2.33 | 0.07 | −2.27 | $1.30 \times 10^{-2}$ |
| Itih3 | chr14:30908573-30923587 | 1.9 | −0.4 | −2.3 | $1.30 \times 10^{-2}$ |
| Cuzd1 | chr7:131308553-131322292 | 3.96 | 1.52 | −2.44 | $1.30 \times 10^{-2}$ |
| Spp1 | chr5:104435110-104441053 | 4.65 | 2.14 | −2.51 | $1.30 \times 10^{-2}$ |
| Prom2 | chr2:127526952-127541417 | 1.92 | −0.6 | -2.52 | $1.30 \times 10^{-2}$ |
| Ttr | chr18:20665249-20674326 | 4.93 | 2.27 | −2.66 | $1.30 \times 10^{-2}$ |
| Hpd | chr5:123171806-123182686 | 3.06 | 0.35 | −2.71 | $2.21 \times 10^{-2}$ |
| Apoh | chr11:108395296-108414396 | 3.46 | 0.68 | −2.79 | $1.30 \times 10^{-2}$ |
| Gnmt | chr17:46725663-46729165 | 3.28 | 0.45 | −2.83 | $1.30 \times 10^{-2}$ |

| | | | | | |
|---|---|---|---|---|---|
| Hpx | chr7:105591610-105600116 | 4.13 | 1.25 | −2.88 | $1.30 \times 10^{-2}$ |
| Fabp1 | chr6:71199887-71205023 | 6.41 | 3.49 | −2.93 | $1.30 \times 10^{-2}$ |
| Mug1, Mug2 | chr6:121781824-122085967 | 2.92 | −0.08 | −3 | $2.21 \times 10^{-2}$ |
| Cyp2c29, Cyp2c39 | chr19:39287084-39568529 | 2.85 | −0.29 | −3.14 | $1.30 \times 10^{-2}$ |
| Cyp2c70 | chr19:40153360-40187286 | 2.9 | −0.35 | −3.24 | $1.30 \times 10^{-2}$ |
| Itih2 | chr2:10094590-10130683 | 1.93 | −1.37 | −3.3 | $1.30 \times 10^{-2}$ |
| Apoa1 | chr9:46228629-46230469 | 6.07 | 2.63 | −3.44 | $1.30 \times 10^{-2}$ |
| Arg1 | chr10:24915206-24927470 | 2.95 | −0.51 | −3.46 | $1.30 \times 10^{-2}$ |
| Kng1 | chr16:23058299-23082078 | 2.93 | −0.55 | −3.48 | $1.30 \times 10^{-2}$ |
| Aldob | chr4:49535994-49549483 | 3.72 | 0.23 | −3.49 | $1.30 \times 10^{-2}$ |
| Ambp | chr4:63143278-63154142 | 3.8 | 0.31 | −3.49 | $1.30 \times 10^{-2}$ |
| Pah | chr10:87521794-87584137 | 1.65 | −1.88 | −3.53 | $1.30 \times 10^{-2}$ |
| Slco1b2 | chr6:141629517-141686635 | 1.24 | −2.35 | −3.59 | $4.75 \times 10^{-2}$ |
| Rdh7 | chr10:127884026-127888733 | 2.05 | −1.58 | −3.63 | $1.30 \times 10^{-2}$ |
| Apof | chr10:128267996-128270151 | 1.7 | −1.94 | −3.64 | $4.75 \times 10^{-2}$ |
| Gc | chr5:89417510-89457898 | 4.75 | 1.08 | −3.67 | $1.30 \times 10^{-2}$ |
| Itih1 | chr14:30929179-30943289 | 1 | −2.71 | −3.71 | $3.19 \times 10^{-2}$ |
| Azgp1 | chr5:137981520-137990232 | 2.32 | −1.41 | −3.73 | $4.75 \times 10^{-2}$ |
| Akr1c6 | chr13:4434342-4457530 | 2.87 | −0.87 | −3.74 | $4.75 \times 10^{-2}$ |
| Bhmt | chr13:93616890-93637758 | 3.96 | 0.17 | −3.79 | $1.30 \times 10^{-2}$ |
| Alb | chr5:90460888-90476603 | 8.5 | 4.62 | −3.88 | $1.30 \times 10^{-2}$ |
| Cyp3a11, Cyp3a41a | chr5:145694048-145879854 | 4.16 | 0.27 | −3.89 | $1.30 \times 10^{-2}$ |
| Pzp | chr6:128438756-128526720 | 2.79 | −1.11 | −3.9 | $1.30 \times 10^{-2}$ |
| Plg | chr17:12378608-12419384 | 3.05 | −0.87 | −3.92 | $1.30 \times 10^{-2}$ |
| Rgn | chrX:20549817-20562087 | 2.67 | −1.29 | −3.96 | $1.30 \times 10^{-2}$ |
| Fgg | chr3:83007895-83015049 | 4.71 | 0.74 | −3.97 | $1.30 \times 10^{-2}$ |
| Fgb | chr3:83042304-83049790 | 4.87 | 0.88 | −3.99 | $1.30 \times 10^{-2}$ |
| F2 | chr2:91625319-91636457 | 2.86 | −1.16 | −4.02 | $1.30 \times 10^{-2}$ |
| Gjb1 | chrX:101376375-101385629 | 1.99 | −2.12 | −4.11 | $4.07 \times 10^{-2}$ |
| Apob | chr12:7977676-8016839 | 1.88 | −2.46 | −4.34 | $1.30 \times 10^{-2}$ |

| | | | | | |
|---|---|---|---|---|---|
| Dio3os, Mir1247 | chr12:110275384-110278656 | 4.86 | 0.42 | −4.43 | $1.30 \times 10^{-2}$ |
| Fga | chr3:83026152-83033617 | 4.17 | −0.42 | −4.59 | $1.30 \times 10^{-2}$ |
| Cps1 | chr1:67123026-67231267 | 2.66 | −1.94 | −4.6 | $1.30 \times 10^{-2}$ |
| Mat1a | chr14:41105032-41124428 | 2.96 | −1.67 | −4.62 | $1.30 \times 10^{-2}$ |
| Spint4 | chr2:164698500-164702448 | 0.01 | −10 | −10.01 | $2.21 \times 10^{-2}$ |
| Defb43 | chr14:63011770-63018088 | 0.35 | −10 | −10.35 | $1.30 \times 10^{-2}$ |
| Bsph2 | chr7:13554865-13571067 | 0.43 | −10 | −10.43 | $1.30 \times 10^{-2}$ |
| Rnase12 | chr14:51056697-51057242 | 0.84 | −10 | −10.84 | $1.30 \times 10^{-2}$ |
| Defb37 | chr8:18986232-18991055 | 1.5 | −10 | −11.5 | $1.30 \times 10^{-2}$ |
| AY761185 | chr8:20943693-20944748 | 2.11 | −10 | −12.11 | $1.30 \times 10^{-2}$ |
| Defb28 | chr2:152518254-152521447 | 3.29 | −10 | −13.29 | $1.30 \times 10^{-2}$ |
| Defb38 | chr8:19023463-19026529 | 3.44 | −10 | −13.44 | $2.21 \times 10^{-2}$ |

**Table S2.** GSEA analysis: summary of top enriched gene sets in the gonadal adipose tissue of mice exposed perinatally to 0.2 mg/kg body weight BDE-47.

| NAME | SIZE | Enrichment score | Normalised enrichment score | NOM *p*-val | FDR *q*-val | FWER *p*-val | RANK AT MAX |
|---|---|---|---|---|---|---|---|
| HALLMARK_COAGULATION | 108 | −0.7925364 | −1.7812736 | 0 | 0.004101 | 0.004 | 582 |
| HALLMARK_XENOBIOTIC_METABOLISM | 167 | −0.72691166 | −1.6750236 | 0 | 0.027806 | 0.055 | 446 |
| HALLMARK_BILE_ACID_METABOLISM | 87 | −0.72079074 | −1.5710769 | 0.0035714 | 0.098446 | 0.256 | 366 |
| HALLMARK_COMPLEMENT | 143 | −0.60537505 | −1.3765348 | 0.0503433 | 0.676869 | 0.942 | 2331 |
| REACTOME_SMOOTH_MUSCLE_CONTRACTION | 20 | 0.75713444 | 1.90847 | 0.06639 | 1 | 1 | 253 |
| MEINHOLD_OVARIAN_CANCER_LOW_GRADE_UP | 16 | 0.7323402 | 1.7202928 | 0.1065292 | 1 | 1 | 31 |
| HORTON_SREBF_TARGETS | 20 | 0.7254165 | 1.7181175 | 0.092511 | 1 | 1 | 1471 |
| REACTOME_MUSCLE_CONTRACTION | 30 | 0.6751347 | 1.6108487 | 0.1088083 | 1 | 1 | 456 |
| NADLER_OBESITY_DN | 44 | 0.66954297 | 1.5260774 | 0.0617284 | 1 | 1 | 1884 |
| REACTOME_CHOLESTEROL_BIOSYNTHESIS | 18 | 0.64574134 | 1.5223196 | 0.1439114 | 1 | 1 | 1672 |
| HSIAO_LIVER_SPECIFIC_GENES | 172 | −0.883853 | −2.028348 | 0 | 0 | 0 | 488 |
| CAIRO_LIVER_DEVELOPMENT_DN | 172 | −0.8234503 | −1.926647 | 0 | 0 | 0 | 673 |
| SU_LIVER | 38 | −0.92811877 | −1.9205562 | 0 | 0 | 0 | 429 |
| CHIANG_LIVER_CANCER_SUBCLASS_PROLIFERATION_DN | 129 | −0.8457685 | −1.8965874 | 0 | 0.00178 | 0.007 | 429 |
| OHGUCHI_LIVER_HNF4A_TARGETS_DN | 82 | −0.8383536 | −1.8692034 | 0 | 0.005103 | 0.025 | 968 |
| YAMASHITA_LIVER_CANCER_STEM_CELL_DN | 49 | −0.8607048 | −1.8556718 | 0 | 0.008842 | 0.051 | 404 |

| | | | | | | | |
|---|---|---|---|---|---|---|---|
| KEGG_COMPLEMENT_AND_COAGULATION_CASCADES | 47 | −0.85020536 | −1.8207873 | 0 | 0.036708 | 0.23 | 480 |
| LEE_LIVER_CANCER_SURVIVAL_UP | 118 | −0.80631876 | −1.8192244 | 0 | 0.034545 | 0.243 | 446 |
| HOSHIDA_LIVER_CANCER_SUBCLASS_S3 | 209 | −0.77290404 | −1.80562 | 0 | 0.056649 | 0.399 | 643 |
| BIOCARTA_INTRINSIC_PATHWAY | 20 | −0.89908606 | −1.8001451 | 0.0026631 | 0.063124 | 0.471 | 446 |
| REACTOME_FORMATION_OF_FIBRIN_CLOT_CLOTTING_CASCADE | 22 | −0.8958861 | −1.7939211 | 0 | 0.070832 | 0.548 | 480 |
| KONDO_PROSTATE_CANCER_WITH_H3K27ME3 | 47 | −0.8267031 | −1.7826784 | 0.0011792 | 0.092976 | 0.673 | 682 |
| SERVITJA_ISLET_HNF1A_TARGETS_DN | 64 | −0.82448655 | −1.7759959 | 0 | 0.107639 | 0.758 | 673 |
| LEE_LIVER_CANCER_CIPROFIBRATE_DN | 34 | −0.84275985 | −1.7544557 | 0.004908 | 0.191406 | 0.924 | 550 |
| LEE_LIVER_CANCER | 30 | −0.86145705 | −1.7486897 | 0.0012821 | 0.208157 | 0.948 | 397 |
| GO_BLOOD_MICROPARTICLE | 71 | −0.8602677 | −1.8951263 | 0 | 0.010074 | 0.01 | 272 |
| GO_ACUTE_PHASE_RESPONSE | 24 | −0.8971937 | −1.8471518 | 0 | 0.05451 | 0.101 | 335 |
| GO_SERINE_TYPE_ENDOPEPTIDASE_INHIBITOR_ACTIVITY | 46 | −0.8429749 | −1.8244396 | 0 | 0.105001 | 0.264 | 242 |
| GO_LIPOPROTEIN_PARTICLE_RECEPTOR_BINDING | 16 | −0.9261174 | −1.814157 | 0 | 0.115658 | 0.371 | 272 |
| GO_POSITIVE_REGULATION_OF_COAGULATION | 24 | −0.88361436 | −1.8138926 | 0.001297 | 0.092931 | 0.373 | 480 |
| GO_ACUTE_INFLAMMATORY_RESPONSE | 41 | −0.8442391 | −1.8065432 | 0 | 0.104209 | 0.467 | 457 |
| GO_REGULATION_OF_COAGULATION | 74 | −0.8127593 | −1.785235 | 0 | 0.188057 | 0.728 | 786 |
| GO_BLOOD_COAGULATION_FIBRIN_CLOT_FORMATION | 18 | −0.9093529 | −1.7776664 | 0 | 0.210428 | 0.81 | 480 |
| GO_NEGATIVE_REGULATION_OF_COAGULATION | 42 | −0.84488016 | −1.7738291 | 0.0012438 | 0.211958 | 0.848 | 233 |
